# *PTCH* mutations in basal cell carcinomas from azathioprine-treated organ transplant recipients

**DOI:** 10.1038/sj.bjc.6604665

**Published:** 2008-10-14

**Authors:** C A Harwood, N R Attard, P O'Donovan, P Chambers, C M Perrett, C M Proby, J M McGregor, P Karran

**Affiliations:** 1 Centre for Cutaneous Research and Department of Dermatology, Institute of Cell and Molecular Science, Bart's and The London, Queen Mary School of Medicine and Dentistry, 4 Newark Street, London E1 2AT, UK; 2 Mammalian DNA Repair Laboratory, Cancer Research UK, London Research Institute, Clare Hall Laboratories, Blanche Lane, South Mimms, Hertfordshire EN6 3LD, UK; 3 Cancer Research UK Mutation Detection Facility, St James's University Hospital, Leeds LS9 7TF, UK

**Keywords:** azathioprine, basal cell carcinoma, PTCH gene, immunosuppression, organ transplant recipients

## Abstract

The immunosuppressant azathioprine is used to prevent graft rejection after organ transplantation. To investigate whether azathioprine-associated mutagenesis contributes to the high incidence of skin tumours in organ transplant recipients (OTRs), we analysed *PTCH* gene mutations in 60 basal cell carcinomas (BCC); 39 from OTRs receiving azathioprine and 21 from individuals never exposed to azathioprine. *PTCH* was mutated in 55% of all tumours, independent of azathioprine treatment. In both the azathioprine and non-azathioprine groups, transitions at dipyrimidine sequences, considered to indicate mutation by ultraviolet-B radiation, occurred frequently in tumours from chronically sun-exposed skin. In BCC from non-sun-exposed skin of azathioprine-treated patients, there was an over-representation of unusual G:C to A:T transitions at non-dipyrimidine sites. These were exclusive to the azathioprine-exposed group and all in the same TGTC sequence context at different positions within *PTCH*. Meta-analysis of 247 BCCs from published studies indicated that these mutations are rare in sporadic BCC and had never previously been reported in this specific sequence context. This study of post-transplant BCC provides the first indication that azathioprine exposure may be associated with *PTCH* mutations, particularly in tumours from non-sun-exposed skin.

Keratinocyte skin cancer (KSC) is the most common form of malignancy following solid organ transplantation. Up to 70% of fair-skinned solid organ transplant recipients (OTRs) will eventually develop KSC of whom two-thirds will have multiple tumours (Euvrard *et al*, 2003). Among these patients, the incidence of squamous cell carcinoma (SCC) is >100-fold higher than in the general population. They also have a significantly elevated (>10-fold) risk of basal cell carcinoma (BCC) (Euvrard *et al*, 2003). Epidemiological and limited molecular data implicate exposure to solar ultraviolet radiation (UVR) and the duration of immunosuppressive treatment in the development of transplant-related KSC (McGregor *et al*, 1997; Euvrard *et al*, 2003; Queille *et al*, 2007). The ultraviolet-B (UVB, 280–320 nm) component of UVR is a known mutagen that causes transition mutations predominantly at dipyrimidine sequences (Brash *et al*, 1991). It also activates signalling pathways that enhance selection of neoplastic cells as well as suppressing immunity (de Gruijl, 1999; Dlugosz *et al*, 2002; Ullrich, 2005). Some commonly used immunosuppressants are carcinogens (IARC, 1987, 1990), although the extent to which impaired immune surveillance *per se* and long-term exposure to carcinogenic immunosuppressant drugs contribute to KSC in OTR is unknown.

The thiopurine pro-drug azathioprine (Aza) is a widely used immunosuppressant. Azathioprine causes 6-thioguanine (6-TG) to be incorporated into DNA where it can become methylated to produce a potentially promutagenic DNA lesion (Swann *et al*, 1996) or interact with UVA (320–400 nm). We have previously shown that, in the latter photochemical reaction, biologically relevant doses of UVA combine with DNA 6-TG and molecular oxygen to generate mutagenic reactive oxygen species and at least one novel and potentially promutagenic DNA lesion, guanine-6-sulphonate (GSO_3_) (O’Donovan *et al*, 2005; Karran and Attard, 2008). The combination of DNA 6-TG/UVA is mutagenic in cultured cells (O’Donovan *et al*, 2005). Photochemical DNA 6-TG damage also occurs in the clinical situation, and the skin of patients taking azathioprine is selectively hypersensitive to UVA (O’Donovan *et al*, 2005; Perrett *et al*, 2008). On the basis of these findings, we now examine the hypothesis that azathioprine may contribute directly to post-transplant cutaneous carcinogenesis.

Molecular analysis in KSC has largely centred on the *TP53* gene, in which characteristic UVB signature mutations have been reported in up to 90% of sporadic SCC (Ziegler *et al*, 1994) and 60% of BCC (Ponten *et al*, 1997). Ultraviolet-B signature mutations have also been identified in the *PTCH* gene in BCCs (Gailani *et al*, 1996; Wolter *et al*, 1997; Lindstrom *et al*, 2006; Heitzer *et al*, 2007), and the mutational inactivation of *PTCH*, rather than *TP53*, is likely to be rate limiting for BCC. The *PTCH* tumour suppressor gene is located on chromosome 9q22, and germline mutations are found in the autosomal dominant condition nevoid BCC (Gorlin) syndrome (NBCCS, MIM 109400), in which patients typically develop numerous BCCs, usually from an early age (Hahn *et al*, 1996; Johnson *et al*, 1996). *PTCH* is also mutated in up to 70% of sporadic BCC (Gailani *et al*, 1996; Hahn *et al*, 1996; Johnson *et al*, 1996; Unden *et al*, 1996; Wolter *et al*, 1997; Aszterbaum *et al*, 1998; Ratner *et al*, 2001; Zhang *et al*, 2001; Kim *et al*, 2002; Reifenberger *et al*, 2005; Teh *et al*, 2005; Heitzer *et al*, 2007). Germline *PTCH* mutations in NBCCS are distributed throughout the entire gene and the majority cause protein truncation (Wicking *et al*, 1997). In BCC from NBCCS, the remaining *PTCH* allele is deleted or inactivated by mutation (Gailani *et al*, 1996). Up to 70% of acquired *PTCH* mutations are UVB-type G:C to A:T transitions or tandem mutations at dipyrimidine sequences (Lindstrom *et al*, 2006; Heitzer *et al*, 2007).

*PTCH* encodes a component of Hedgehog (HH) signalling, a fundamental developmental signal transduction pathway that also controls epidermal cell proliferation. *PTCH* comprises 23 exons spanning approximately 50 kb and encodes a 1447-amino acid 12-transmembrane domain glycoprotein that binds Sonic hedgehog (SHH), a secreted ligand regulating proliferation and patterning of multiple tissues during embryogenesis. Current models propose that PTCH inhibits HH signalling through interaction with the Smoothened (SMOH) protein (Bale and Yu, 2001) thereby repressing expression of the Gli1 and Gli2 transcription factors, which are themselves key mediators of HH signalling (Dahmane *et al*, 1997; Regl *et al*, 2002). PTCH repression of SMOH is relieved upon binding of SHH to PTCH resulting in the activation of Gli1 and Gli2 and several other genes including, for example, *TGF-beta*, *Wnt*, *BCL-2* (Regl *et al*, 2002), *basonuclin* (Cui *et al*, 2004) and the forkhead transcription factors *FOXMI* (Teh *et al*, 2002) and *FOXE1* (Eichberger *et al*, 2004). Temporally and spatially constrained SHH signalling regulates cyclic growth of hair follicle epithelium, but mutational inactivation of *PTCH*, or activating mutations in *SMOH* or *SHH,* leads to constitutive HH signalling (Unden *et al*, 1996; Oro *et al*, 1997; Xie *et al*, 1998; Bale and Yu, 2001). The resulting sustained proliferation drives the development of BCC and other follicle-derived tumours.

To investigate the role of azathioprine in the development of transplant-related BCC, we compared the *PTCH* mutation spectrum in sporadic BCC from patients immunosuppressed with azathioprine to the spectrum from individuals not exposed to azathioprine. The relative genetic homogeneity of BCC makes it a more tractable experimental system for molecular analysis than the genetically heterogeneous SCC (Quinn *et al*, 1994; Ashton *et al*, 2005; Purdie *et al*, 2007). In addition, the potentially rate-limiting nature of *PTCH* inactivation is likely to be relevant to key mutational events in BCC. In this first study of post-transplant BCC, we report differences in the *PTCH* mutational spectrum that we propose might reflect a direct influence of azathioprine exposure on BCC development.

## Materials and methods

### Patients and samples

Our institution has a dedicated OTR skin clinic in which over 1000 OTRs have been under surveillance since 1989 (Harwood *et al*, 2006; Ismail *et al*, 2006). Patients are seen within 12 months of transplantation and at least annually thereafter. Basal cell carcinomas from azathioprine-exposed individuals were obtained from this patient group. Thiopurine methyltransferase and thioguanine nucleotide levels, although useful for predicting acute myelotoxicity, were not specifically included in this study because all patients had been on azathioprine for at least 6 months and had stable haematological parameters. Non-azathioprine-exposed individuals were recruited from among immunocompetent (IC) individuals attending general dermatology clinics at the same institution. Clinical details including duration of transplantation, immunosuppressive drug history and site of BCC were documented.

Basal cell carcinomas were defined as sun-exposed (SE) lesions if they arose on chronically SE sites (head, neck, hands and forearms). Other sites not chronically SE or only intermittently SE were defined as non-sun exposed (NSE) and included locations such as trunk and proximal limbs. Basal cell carcinomas on NSE are more common in OTRs, accounting for up to one-third of OTR BCC compared with less than one-fifth of IC BCC (Kanitakis *et al*, 2003; Harwood *et al*, 2006).

All 60 tumours were histologically confirmed. Tumour tissue was collected at the time of surgery, immediately snap frozen and stored at −70°C. DNA was extracted by a standard phenol–chloroform–isoamyl alcohol technique followed by ethanol precipitation. Matched whole blood samples were obtained for 32 tumours and DNA was extracted by Nucleon DNA extraction kit (Scotlab, Lanarkshir, UK).

Ethical approval for this study was obtained from the East London and City Health Authority Ethics Committee. All patients provided written informed consent. The study was conducted according to the Declaration of Helsinki Principles.

### Mutation detection

An initial mutation screening of exons 2–23 of *PTCH* was performed by melting curve analysis (MCA). This study used MCA as a primary mutation screening technique before DNA sequencing. This technique has earlier been shown to be a highly sensitive and specific method for mutation screening (Reed and Wittwer, 2004). Melting curve analysis generates qualitative data; therefore, the nature and locations of mutations was confirmed by DNA sequencing.

PCR was performed in the presence of LCGreen+, a double-stranded DNA-binding dye. PCR products were then melted on a LightScanner (Idaho Technologies, Salt Lake City, UT, USA). As the DNA melted, LCGreen+ was released and there was a reduction in the level of fluorescence. Measurement of differences in the rate of change of fluorescence allowed detection of variations. Melting curve analysis results were scored by two independent scientists. Exon 1B was not screened as the high G+C content of this exon did not permit reliable analysis. Amplification and analysis of exons 2 and 23 in two fragments resulted in the generation of less complex melting profiles and therefore increased the sensitivity of MCA for these exons.

PCRs for MCA contained PCR buffer, 200 *μ*M dNTPs, MgCl_2_ as required (Supplementary Table 1 online), 1 *μ*l LCGreen+ (all Idaho Technologies), 0.25 U of ThermoStart Taq polymerase (ABgene, Epsom, UK), primers as required, DMSO to a final concentration of 5%, 20 ng of genomic DNA and sufficient water to make a final volume of 10 *μ*l. Thermal cycling conditions were 95°C for 12 min to activate the Taq polymerase followed by 45 cycles of 94°C for 10 s, annealing temperature (Supplementary Table 1 online) for 15 s and 72°C for 15 s. To ensure maximum incorporation of LCGreen+ into double-stranded amplified DNA, PCR products were heated to 94°C for 30 s and allowed to cool to 25°C at 0.1°C per second. PCR primer sequences and amplification conditions for each amplified fragment are shown in Supplementary Table 1 online. Variations detected by MCA were then fully characterised by DNA sequencing with a version 1.1 BigDye Terminator kit (Applied Biosystems, Warrington, UK) and analysed on an Applied Biosystems 3730.

DNA and protein numbering was based on the following GenBank accessions: genomic DNA AL161729, cDNA NM_000264 and protein NP_000255. Mutations and single-nucleotide polymorphisms were compared with the GenBank database, the PTCH Mutation Database (www.cybergene.se/PTCH/) and relevant publications not yet entered on the *PTCH* Mutation Database at the time of this analysis (specifically Heitzer *et al* (2007)). The effect of intronic mutations on splicing was predicted using software at http://www.fruitfly.org/seq_tools/splice.html. Effect of exonic mutations on exonic splice enhancers was predicted using software at http://rulai.cshl.edu/tools/ESE/

### Statistical analysis

Statistical analysis was performed in Stata (Stata Corp 2003 Statistical Software: Release 9.0: College Station, TX, USA). Formal comparisons were made using logistic regression where possible. Multiple samples from the same individual could not be considered independent. Model-based variance estimates were therefore inappropriate, and robust sandwich estimates of variance were used instead. For comparisons of proportions of those with the unique G:C to A:T transitions at non-dipyrimidine sites, the numbers were too small to allow the use of logistic regression. In this case, and others where numbers were not sufficient, the Fisher's exact test was used. To preserve independence, we used Fisher's exact test on the number of individuals rather than on the number of samples.

## Results

### Patients and tumours

Sixty BCCs from 58 individuals (38 OTRs and 20 IC individuals) were analysed in this study. Detailed patient information is presented in Table 1.

- Azathioprine-exposed individuals (Aza group) comprised 37 OTRs with BCC (30 males, 7 females). The mean age at sampling was 58.8 years (38.6–79.0 years) and mean duration of transplantation was 134 months (range 6–296 months). Thirty-two individuals were currently receiving systemic immunosuppression with azathioprine and five had previously received azathioprine, before BCC diagnosis. Additional immunosuppressants included ciclosporin (*n*=24) and prednisolone (*n*=36). Twenty of these azathioprine-exposed OTRs had a previous history of KSC, 12 out of 37 (32.4%) with at least one BCC.
- Non-azathioprine-exposed subjects (non-Aza group) included 20 IC individuals (13 men, 7 women; mean age 73.9 years) and one OTR (males; age 69.4 years; duration of transplant 127 months), never exposed to azathioprine. This OTR had three prior BCCs but none of the IC individuals had a history of KSC.

Overall, therefore, 39 tumours were from the Aza group in which two patients each had two separate tumours. There were 21 tumours from non-Aza individuals. The 60 BCCs comprised 50 nodular, 8 superficial and 2 infiltrative/morphoeic subtypes.

### Azathioprine exposure and the frequency and location of PTCH mutations

There were no germline *PTCH* mutations in any of the individuals examined. *PTCH* mutations were detected in 33 out of 60 (55%) BCC overall. Azathioprine exposure did not influence this frequency. A total of 17 mutations were found in 11 out of 21 (52%) non-azathioprine-exposed BCCs (Table 2) *vs* 27 mutations from 21 out of 39 (54%) azathioprine-exposed BCCs (Table 3).

The proportion of exonic and intronic mutations was similar in the two groups; intronic mutations accounted for 6 out of 17 (35%) mutations in the non-Aza group and 9 out of 27 (33%) mutations in the Aza group (Table 4). Analysis of mutation distribution over the predicted PTCH protein structure (www.cybergene.se/PTCH/) identified no significant differences between the Aza and non-Aza groups. Extracellular loop domains were the most commonly mutated sites in both the groups accounting for 13 out of 18 (72%) exonic mutations in the Aza group and 6 out of 11 (55%) in the non-Aza group. Table 4 summarises the distribution of mutations on the PTCH protein domains.

Nonsense and stop mutations were the most frequently observed *PTCH* mutations, together accounting for 8 out of 17 (47.1%) and 15 out of 27 (55.6%) mutations in non-Aza *vs* Aza group, respectively.

### PTCH mutation spectra in BCC from azathioprine- and non-azathioprine-exposed patients

#### PTCH mutations in azathioprine-exposed patients

**Figure 1:**
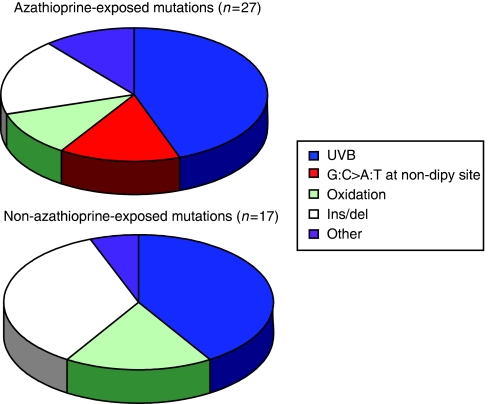
PTCH mutation spectra in BCC from azathioprine- and non-azathioprine-treated patients. A similar proportion of UVB and oxidation-type mutations is present in each group. The G:C to A:T transition mutations at non-dipyrimidine sites (hatched) occured exclusively in the azathioprine-exposed group. These were all within the same TGTC sequence in different locations within PTCH.

**Figure 2:**
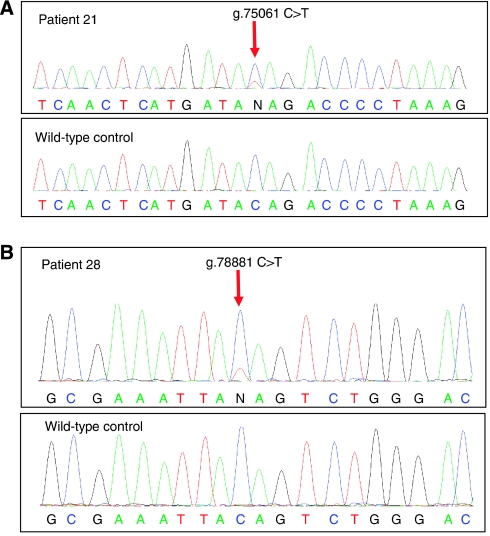
Sequencing data in BCC from azathioprine-exposed individuals. (**A**) Mutation in PTCH exon 3 (g.75061 C>T, c.478 C>T, p. Q160X). Data from patient BCC ID 21 (top) and from a wild-type control (bottom). (**B**) Mutation in PTCH exon 5 (g.78881 C>T, c. 724 C>T, p. Q242X). Sequencing data are from patient BCC ID 28 (top) and from a wild-type control (bottom).

*PTCH* mutations were detected in 21 out of 39 (54%) BCCs from OTRs exposed to azathioprine (Table 3). Seventeen (81%) had a single mutation and the remainder had between two and four mutations each. Of the 27 individual mutations, there were 12 (44.4%) transitions at dipyrimidine sites, consistent with UVB involvement. A further three mutations (11.1%) were G:C to T:A transversions generally regarded to reflect oxidative damage in the form of unrepaired DNA 8-oxoguanine. There were five (18.5%) deletions or insertions, all at non-repetitive sequences. We also observed four (14.8%) G:C to A:T transitions at non-dipyrimidine sites (Figure 1). These unusual mutations occurred in samples 21, 28, 38 and 57 from three patients who were receiving only prednisolone and azathioprine. Strikingly, although each mutation was at a different locus in the *PTCH* gene in each individual, they shared an identical TGTC sequence context (Figure 2). Two of these mutations occurred in apparently independent superficial BCCs (samples 28 and 57, Table 3) that developed in close proximity on the trunk of a single patient. It is possible that these were, in fact, contiguous regions of a single superficial tumour, the entirety of which was not clinically apparent. Nevertheless, we have considered these clinically distinct tumours to be of independent origin.

#### PTCH mutations in non-azathioprine-exposed patients

*PTCH* mutations were identified in 12 out of 21 (57.1%) BCCs from patients never treated with azathioprine (20 IC individuals and one OTR) (Table 2). Seven of these tumours (58.3%) contained a single mutation and five each contained two. Of the 17 individual mutations, seven (41.2%) UVB signatures at dipyrimidine sites comprised the largest single group. Three (17.6%) were G:C to T:A transversions, indicating a similar frequency of oxidation-related mutations as in the Aza group. There were six (35.3%) deletions of between 1 and 10 bases in moderately repetitive or non-repetitive sequences. We did not observe any of the G:C to A:T transitions at non-dipyrimidine sites that were found in the Aza group (Figure 1).

To examine the possible cumulative effects of azathioprine treatment on mutation type, the Aza-treated group was divided into two subgroups, having received greater or less than 120 months of azathioprine treatment. Of 15 mutations in the group exposed for less than 120 months, 8 were UVB type. Among those more extensively exposed (>120 months) there were 4 (of 12). The difference in UVB-type mutations between the two groups is not statistically significant (*P*-value=0.571; adjusted for sun exposure, sex and clustering within individuals). Three of the four G:C to A:T transition mutations at non-dipyrimidine sites, exclusive to the azathioprine-exposed group, were in patients exposed for >120 months.

### The effect of additional immunosuppressants

PTCH mutations were equally frequent in BCCs from patients exposed to azathioprine and prednisolone (7 out of 15, 47% BCC) and in the group treated with ciclosporin in addition to azathioprine and prednisolone (14 out of 24, 58.3% BCC). At the time of tumour removal, two patients in the ciclosporin and Aza group had discontinued prednisolone. The majority of mutations in the group exposed to all three immunosuppressants were of the UVB type (11 out of 19, 57.9% individual mutations). In the azathioprine plus prednisolone group, only one of eight (12.5%) mutations was a UVB type and this group contained all four of the G:C to A:T transitions at non-dipyrimidine sites. The apparently lower frequency of UVB-type mutations in the azathioprine/prednisolone group may simply reflect the imbalance between SE and NSE BCCs in the two groups as six (of eight) individual mutations in the azathioprine/prednisolone group were from BCCs on NSE sites.

### PTCH mutations and sun exposure

Although the involvement of UVB mutagenesis in *PTCH* of BCCs from IC individuals has previously been reported, nothing is known about the role of UVB in BCC from azathioprine-treated patients. To address the latter point, we specifically examined PTCH mutations in BCCs from chronically SE sites. In addition, as BCCs are more common on NSE skin in OTR than in IC individuals, it is plausible that the effects of immunosuppressants are more apparent in the absence of chronic UVB exposure. To increase the chances of detecting azathioprine-related mutations, we therefore chose to examine more NSE BCC from the Aza group (the ratio of SE to NSE tumours in the Aza group was approximately 1 : 1 compared with 4 : 1 in the non-Aza group).

Of the 60 BCCs analysed, 37 had occurred on SE sites. Of these, 20 were from azathioprine-treated patients and 17 from non-Aza patients. *PTCH* mutations occurred at similar frequencies in the two groups (60 and 70.6%, respectively). As expected, UVB mutations were the largest single mutational class in the non-Aza BCC and one or more mutations of this type were found in 6 out of 12 (50%). Ultraviolet-B mutations were also the predominant class in azathioprine-treated patients and were found in 9 out of 12 (75%) BCC in this group.

There were only four NSE BCCs from the IC patients, none of which contained a mutated *PTCH*. Of the 37 BCCs in the azathioprine-treated group, 19 were from NSE skin. Nine (47.4%) of these NSE BCCs contained a *PTCH* mutation but only one was a UVB type. In contrast, C to T transitions at non-dipyrimidine sites in a TGTC sequence comprised almost half (four out of nine) of the mutations in this azathioprine-treated group. The significant (*P*=0.02) over-representation of these mutations in NSE BCC suggests that they are unrelated to sunlight exposure and this route to *PTCH* inactivation is more likely in BCC on NSE skin of azathioprine-treated patients in which the contribution of UVB is minimised.

## Discussion

To our knowledge, *PTCH* mutations in BCCs from immunosuppressed OTR have not previously been systematically investigated. *PTCH* was mutated in around half (55%) of the BCCs examined, and this was independent of immune status and azathioprine treatment. This frequency is comparable with the known *PTCH* mutation frequency in BCCs from the general population. A recent comprehensive study (Heitzer *et al*, 2007) found *PTCH* to be mutated in 29 out of 60 tumours (48%). We carried out a meta-analysis of 11 studies in BCCs from presumed IC individuals published between 1996 and 2007 (Gailani *et al*, 1996; Hahn *et al*, 1996; Johnson *et al*, 1996; Unden *et al*, 1996; Wolter *et al*, 1997; Aszterbaum *et al*, 1998; Ratner *et al*, 2001; Zhang *et al*, 2001; Kim *et al*, 2002; Reifenberger *et al*, 2005; Heitzer *et al*, 2007). This revealed that 105 out of 247 (42.5%) tumours had at least one defined PTCH mutation. It appears that *PTCH* mutation occurs at a similar high frequency in BCCs from both IC and immunosuppressed individuals.

The contribution of high-intensity sunlight exposure to BCC development in the general population is well established (Pelucchi *et al*, 2007). Compelling evidence for the involvement of UVB radiation comes from the prevalence of transitions at dipyrimidine sites that are generally regarded as signature UVB mutations. Heitzer *et al*, report that almost 70% of exonic *PTCH* mutations in sporadic BCC were of this type. This incidence of UVB signature mutations is somewhat higher than the frequencies reported in other studies. Our meta-analysis, while confirming the high frequency of *PTCH* mutations, revealed that UVB-type mutations were present in half (51.4%) of the BCCs. Our analysis of BCC from immunosuppressed OTR demonstrated that UVB signatures were the predominant mutational class and comprised almost half of all *PTCH* mutations in the azathioprine-exposed (44.4%) group. The frequency was the same (41.2%) in the non-azathioprine-treated control group. These combined data affirm the relative genetic homogeneity of sporadic BCC of diverse clinical phenotype, the predominant causative role of UVB-induced DNA damage and the relatively minor contribution of oxidative DNA damage.

There are differences in the anatomical distribution of BCCs in immunosuppressed OTRs and those in the general population. Both develop head and neck lesions, but OTRs also have BCCs on relatively NSE skin, particularly the trunk and limbs (Kanitakis *et al*, 2003; Harwood *et al*, 2006). This has previously been attributed, at least in part, to ascertainment bias as OTRs are regularly systematically examined in dedicated transplant clinics. However, it may reflect the presence of another carcinogen that would be supported by the PTCH mutation analysis in this series.

The frequency of *PTCH* mutations in BCCs from NSE skin of azathioprine-treated patients was similar to that in BCC overall, but there were quantitative and qualitative differences in the mutational spectrum that we observed. UV-type mutations were under-represented, consistent with a lesser role for UVB on sites receiving either no, or only intermittent, sun exposure. In addition, we found an unusual class of apparently sequence-related non-dipyrimidine site transitions in some of these OTR BCCs that could represent a mutational footprint associated with immunosuppressant (presumed Aza) treatment. We acknowledge that in addition to azathioprine, all patients in the organ transplant group had also been treated with prednisolone and, in many cases, ciclosporin. There is little evidence that corticosteroids are mutagenic (IARC Monographs: Prednisolone, 1987) but ciclosporin is a recognised human carcinogen (IARC Monographs: Ciclosporin, 1990). We note, however, that the spectrum of *PTCH* mutations in BCCs from our patients taking ciclosporin is closely similar to that for BCCs in non-immunosuppressed patients.

Our meta-analysis of 247 sporadic BCCs from patients not receiving azathioprine compared with our own findings suggests that the frequency of the non-dipyrimidine site transition is increased by azathioprine treatment. It was present in 4 out of 266 *vs* 3 out of 37 individuals in the non-Aza and Aza groups, respectively (*P*=0.042; Fisher's exact test). In addition, the unusual class of mutations was found in patients whose BCCs had developed after prolonged Aza exposure, suggesting that it may be dose related.

Azathioprine, and the related 6-mercaptopurine and 6-TG, have been in clinical use for more than 50 years. They are not powerful mutagens or carcinogens, although the incorporation of 6-TG in place of G in DNA is potentially weakly promutagenic. Substantial substitution of DNA G by 6-TG is well tolerated by cells and DNA 6-TG is not significantly miscoding during replication. The -SH group of 6-TG is, however, chemically reactive and cellular DNA 6-TG can become methylated or oxidised. Although likely to be produced only at low levels, the products of these chemical reactions – S^6^-methyl-TG (S^6^-meTG) and GSO_3_ – are potentially highly mutagenic. The former miscodes during replication and directs the incorporation of A–a promutagenic change. Guanine-6-sulphonate is a severe block to replication and may require the recruitment of error-prone DNA polymerases to effect bypass (O’Donovan *et al*, 2005; Zhang *et al*, 2007).

In cultured cells, 6-TG/UVA treatment is associated with an increased frequency of the G:C to C:G transversions (O’Donovan, 2006) consistent with oxidation (Henderson *et al*, 2003). As keratinocytes from patients treated with azathioprine contain measurable DNA 6-TG and their skin is selectively UVA sensitive (Perrett *et al*, 2008), we anticipated an increase in oxidation-type *PTCH* mutations in BCC from azathioprine-treated patients. We did not observe this. The reasons for this are unclear, but a contributory factor may be the relative anoxia of keratinocytes compared with cultured cells.

This study of *PTCH* mutation in BCC from azathioprine-treated organ transplant patients and IC individuals confirms that *PTCH* is a major target for inactivation of diverse clinical types in BCC. The findings demonstrate that the major contribution of UVB radiation to the inactivation of *PTCH* in BCC on chronically SE skin is also evident in azathioprine-immunosuppressed patients. Our data also raise the novel possibility of a different aetiology for azathioprine-related BCC developing on areas of skin that generally receive intermittent or no exposure to sunlight. Basal cell carcinomas arising from NSE skin occur infrequently in IC patients (Kanitakis *et al*, 2003) and our panel of BCC included only four examples. None of these contained a *PTCH* mutation that precluded direct comparison of mutations between IC and azathioprine-treated patients. Furthermore, in our meta-analysis of BCC from IC patients, the anatomical site of the BCC was frequently not identified and the number of mutations from BCC on unequivocally NSE sites was again too small (*n*=10) to allow meaningful comparison. It will be important for the significance of future studies to ensure that sufficient samples from IC patients are included.

It seems appropriate to follow up the hypothesis that azathioprine contributes to mutation in BCC from NSE areas with a more extensive study. The *TP53* gene represents an alternative to *PTCH* as a mutational target. *TP53* is mutated in many sporadic BCCs and a large majority of reported mutations are UVB type (Brash *et al*, 1991; Giglia-Mari and Sarasin, 2003; Reifenberger *et al*, 2005). The IARC TP53 Somatic Mutation database lists more than 1000 mutations from normal, preneoplastic and neoplastic skin (Petitjean *et al*, 2007) although an association with immunosuppression is noted in only 26 cases for which the medication is not specified. We are unable to locate any publications that specifically address *TP53* mutation spectra directly in relation to azathioprine exposure. In view of our findings with *PTCH*, it now seems fitting to examine whether promutagenic effects of azathioprine contribute to *TP53* mutation in BCC in areas of the skin that are protected from sunlight.

## Figures and Tables

**Table 1:** Clinical details of patients and tumours

**BCC ID**	**Sex**	**Age (years)** a	**Immune status**	**Aza** b	**Duration of transplant/ Aza exposure (months)** c	**Other IS drugs**	**Location**	**SE/NSE** d	**BCC subtype**
2	M	61.2	IC	N	—	—	Back	NSE	Nod
4	F	72.4	IC	N	—	—	Thigh	NSE	Nod
6	M	67.2	IC	N	—	—	Leg	NSE	Nod
10	M	49.2	IC	N	—	—	Back	NSE	Nod
1	F	81.8	IC	N	—	—	Temple	SE	Nod
3	M	83.2	IC	N	—	—	Ear	SE	Nod
5	M	57.4	IC	N	—	—	Temple	SE	Nod
7	F	68.4	IC	N	—	—	Forehead	SE	Nod
8	F	83.9	IC	N	—	—	Nose	SE	Nod
11	M	87.2	IC	N	—	—	Forearm	SE	Nod
12	F	83.5	IC	N	—	—	Cheek	SE	Nod
13	M	73.3	IC	N	—	—	Nose	SE	Nod
14	M	72.6	IC	N	—	—	Upper lip	SE	Nod
15	M	82.5	IC	N	—	—	Temple	SE	Nod
16	F	89.2	IC	N	—	—	Chin	SE	Nod
17	M	82.5	IC	N	—	—	Cheek	SE	Nod
18	M	85.9	IC	N	—	—	Cheek	SE	Nod
19	F	75.0	IC	N	—	—	Forehead	SE	Inf
20	M	59.6	IC	N	—	—	Nose	SE	Nod
9	M	61.3	IC	N	—	—	Temple	SE	Nod
58	M	59.1	OTR	N	127/−	P,C	Temple	SE	Nod
21	M	52.5	OTR	Y	137	P	Shoulder	NSE	Sup
24	M	47.9	OTR	Y	247	P	Shoulder	NSE	Sup
27	M	59.4	OTR	Y	247	P	Chest	NSE	Nod
28^e^	M	54.8	OTR	Y	171	P	Mid-back	NSE	Sup
31	M	42.9	OTR	Y	239	P	Shoulder	NSE	Nod
32	M	57.6	OTR	Y	24	P,C	L Shin	NSE	Nod
35	F	62.9	OTR	Y	296	P	Thigh	NSE	Sup
37	M	47.7	OTR	Y	200	P	Flank	NSE	Nod
38	M	48.2	OTR	Y	68	P	Lower leg	NSE	Nod
39	M	49.0	OTR	Y	175	P,C	Upper back	NSE	Sup
40	M	59.8	OTR	Y	155	C	Back	NSE	Nod
49^f^	M	63.2	OTR	Y	104	P,C	Scapula	NSE	Nod
51	M	53.7	OTR	Y	108	P,C	Lower back	NSE	Sup
55	M	46.6	OTR	Y	135	P,C	Shoulder	NSE	Nod
56	F	38.6	OTR	Y	113	P,C	Shoulder	NSE	Nod
57^e^	M	54.8	OTR	Y	171	P	Shoulder	NSE	Sup
59	F	66.0	OTR	Y	86	P	Elbow	NSE	Nod
60	M	56.8	OTR	Y	28	P,C	Mid-back	NSE	Nod
22	M	71.2	OTR	Y	56	P,C	Temple	SE	Nod
23	F	65.6	OTR	Y	78	P,C	Forehead	SE	Nod
25	M	58.7	OTR	Y	212	P	Forearm	SE	Nod
26	M	58.1	OTR	Y	65	P	Forehead	SE	Nod
33	M	79.0	OTR	Y	68	P,C	Post-auricular	SE	Nod
36	F	70.0	OTR	Y	135	C	Forehead	SE	Nod
41	F	68.2	OTR	Y	6	P,C	Chin	SE	Nod
43	M	65.4	OTR	Y	75	P.C	Forehead	SE	Nod
44	M	70.9	OTR	Y	111	P,C	Ear	SE	Inf
46	M	67.4	OTR	Y	100	P,C	Temple	SE	Nod
48^f^	M	63.2	OTR	Y	104	P,C	Cheek	SE	Nod
52	M	65.6	OTR	Y	269	P	Dorsum hand	SE	Nod
53	M	74.8	OTR	Y	189	P	Nose	SE	Nod
54	M	54.6	OTR	Y	99	P,C	Nose	SE	Nod
30	M	53.0	OTR	Y	84	P,C	Neck	SE	Nod
34	M	51.3	OTR	Y	133	P,C	Nose	SE	Sup
29	M	51.1	OTR-F	Ex	168/168	P,C	Temple	SE	Nod
42	M	49.6	OTR-F	Ex	156/156	P	Scapula	NSE	Nod
45	M	66.3	OTR	Ex	227/84	P, C	Chin	SE	Nod
47	M	54.5	OTR	Ex	192/48	P,C	Neck	SE	Nod
50	M	71.2	OTR	Ex	141/114	P,C	Ear	SE	Nod

Aza=azathioprine exposure; BCC=basal cell carcinoma; C=ciclosporin; CIS=squamous carcinoma *in situ* (Bowen's disease); Ex=previously on azathioprine; F=female; IC=immunocompetent individual; inf=infiltrative BCC; IS=immunosuppressive; M=male; nod=nodular BCC; NSE=non-sun-exposed site; P=prednisolone; sample ID=sample identification number; SCC=squamous cell carcinoma; SE=chronically sun-exposed site; sup=superficial BCC; OTR=organ transplant recipient; OTR-F=failed organ transplant.

a Age at removal of BCC (years).

b Azathioprine exposure: Y, currently exposed; Ex, previously exposed to azathioprine, but not at the time of BCC removal; pt 29, transplant failed and off azathioprine for 3 years before removal of BCC (but maintained on ciclosporin and prednisolone); pt 45, off azathioprine for more than 10 years before removal of BCC; pt 47, off azathioprine for at least 8 years before removal of BCC; pt 50, off azathioprine for at least 2 years before removal of BCC.

c For patients on azathioprine at the time of diagnosis, the duration of transplantation is equivalent to the duration of azathioprine exposure. For patients previously exposed but off azathioprine at the time of BCC removal, the duration of previous azathioprine exposure was approximately 168 months, 84 months, 48 months and 114 months for patient nos. 29, 45, 47 and 50, respectively.

d Anatomical location of a BCC was classified as ‘sun-exposed’ if it arose on a chronically UV-exposed site (head and neck, dorsum hands and forearms). BCCs arising on rarely or only intermittently sun-exposed sites were grouped together as ‘non-sun-exposed’.

e Samples 28 and 57 were from the same individual; although apparently clinically separate, the finding of an identical PTCH mutation raises the possibility that they may represent a single lesion.

f Samples 48 and 49 were from the same individual.

**Table 2:** PTCH mutations in BCC from non-azathioprine-exposed individuals

**BCC ID**	**Exon/intronic position**	**Variation in genomic DNA** a ^,^ b	**Sequence context**	**Variation in protein** c	**PTCH domain**	**Effect** d
3	IVS18+5	g.102844 G>T	tga**g**tgt	—	—	Possible effect on splicing
5	Exon 14	g.91889 G>T	gct**g**agc	p. E680X	ICL3	Nonsense mutation
7	Exon 12	g.84793_84801del	ttc**ccgctctgc**ggg	p. P568_R570 delins R	TM5	3-amino-acid deletion, arginine at position 568
	IVS5-1	g.80263 G>A	gca**g**agg	—	—	mRNA splicing
9	IVS8 and exon 9	g.82662_82677del	acc**acaggtggttcatcag**agt	See below^f^	—	See below^f^
12	Exon 15	g.93597 C>T	cac**cc**ag	p. T807T	ECL2	Silent variant; possible effect on exonic splice enhancer
		g.93598 C>T	**Single tandem mutation**	p. Q808X		Nonsense mutation
14	Exon 3	g.75133 C>T	ctc**c**agg	p. Q184X	ECL1	Nonsense mutation
	Exon15	g.93653 del T	gtt**t**cag	p. F826S fsX4	ECL2	Stop codon at position 829
15	Exon 9	g.82774 del G (homozygous)	cgc**g**tgg	p. V442W fsX14	TM2	Stop codon at position 455
16	Exon 16	98986 A>G	tcg**a**cat	p. D898G	ECL2	Missense mutation; possible effect on exonic splice enhancers
	IVS9+1	g.82798 G>A	atg**g**taa	—	—	RNA splicing
17	Exon 18	g.102581 del G	cga**g**tat	p. E970D fsX25	ECL2	Stop codon at position 994
19	IVS21-27	g.111502 C>T	aca**c**ttt	—	—	See below^e^
	IVS8-6	g.82660 C>A	tga**c**cac	—	—	No effect on splicing predicted
20	Exon 10	g.83283_83287del	ttt**ccttt**aac	p.S494X	ECL3	Stop codon at position 494
58^g^	Exon 12	g.84724 G>A	cag**g**agc	p. G545E	ICL2	Missense, possible effect on exonic splice enhancers
	Exon 13	g.90996 C>T	tat**c**gac	p. R602X	ICL3	Nonsense mutation

bp=base pair; del=deletion; fs=frameshift; ins=insertion; IVS=intron; PTCH domains: ECL1=extracellular loop 1; ECL2=extracellular loop 2; ICL3=intracellular loop 3; TM2=transmembrane 2; TM5=transmembrane 5.

a Mutations are corrected for SNPs (using SNPs from blood samples for BCCs 1–12 and from SNP database for BCCs 13–20 and 58)

b Numbering based on GenBank genomic DNA sequence AL161729.

c Applicable to exonic variants only. Protein numbering based on GenBank accession number NP_000255.

d Effect of intronic mutations on splicing was predicted using software at http://www.fruitfly.org/seq_tools/splice.html; effect of exonic mutations on exonic splicing enhancers was predicted using software at http://rulai.cshl.edu/tools/ESE/.

e Splice-site prediction software failed to detect the wild-type splice acceptor site in intron 21 and, therefore, could not predict the effect of any of these variations on splicing.

f This 16-bp deletion deletes the last 4 bp of intron 8 and the first 12 bp of exon 9. The deletion of 4 bp from intron 8 is predicted to have an effect on splicing. The in-frame deletion of 12 bp of exon 9 deletes four amino acids from the PTCH protein: V406, V407, H408 and Q409.

g Patient no. 58 is an organ transplant recipient never previously exposed to azathioprine.

**Table 3:** PTCH mutations in BCC from azathioprine-exposed patients

**BCC ID**	**Exon/intronic position**	**Variation in genomic DNA** a	**Sequence context**	**Variation in protein** b	**PTCH domain**	**Effect** c
21	Exon 3	g.75061 C>T	tcatgata**c**agaccccta	p. Q160X	ECL1	Nonsense mutation
24	Exon 20	g.101733-101734 ins AC	agcACgtc	p. V1137T fsX3	TM11	Stop codon at position 1139
28	Exon 5	g.78881 C>T	cgaaatta**c**agtctggga	p. Q242X	ECL1	Nonsense mutation
30	Exon 6	g.80350 G>A	gct**g**gga	p. W278X	ECL1	Nonsense mutation
33	Exon 2	g.54438 G>A	gtg**g**gtg	p. W129X	ECL1	Nonsense mutation
34	Exon 14	g.91901 G>T	tcc**g**aga	p. E684X	ICL3	Nonsense mutation
35	IVS 2+1	54446 del G	aag**g**taa	—	—	Splicing
36	Exon 22	g.111703 G>A	tac**g**agg	p. E1242K	C-term	Missense mutation; possible effect on exonic splice enhancers predicted
38	Exon 17	g.101078 C>T	tgactaaa**c**agcgtctgg	p. Q905X	ECL2	Nonsense mutation
39^d^	Exon 15	g.93615 del G	ccc**g**aat	p. N814IfsX16	ECL2	Stop codon at position 829
41	Exon 15	g.93622 C>T	atc**c**agc	p. Q816X	ECL2	Nonsense mutation
44	IVS7-2	g.81703 A>C	tgc**a g**cg	—	—	See below^e^
	IVS7-1	g.81704 G>T	tgc**a g**cg	—	—	See below^e^
48	IVS8-6	g.82660 C>T	tga**c**cac	—	—	No effect on splicing predicted
51	Exon 18	102581 del G	cga**g**tat	p. E970D fsX25	ECL2	Stop codon at position 994
53	Exon 23	g.113735 C>T	tcg**c**cgt	p. A1380V	C-term	Missense mutation; possible effect on exonic splice enhancers predicted
	Exon 10	g.83158 T>G	tca**t**gct	p. Y452X	TM2	Nonsense mutation
54	Exon 3	g.75076 G>T	gaa**g**aag	p. E165X	ECL1	Nonsense mutation
56	IVS11-1	g.84692 G>A	cca**g**gac	—	—	Splicing
	Exon 17	g.101230 C>A	cta**c**atg	p. Y955X	ECL2	Nonsense mutation
	IVS21-11	g.111518 C>T	atc**cc**ct	—	—	See below^f^
	IVS21-10	g.111519 C>T	**Single tandem mutation**	—	—	See below^f^
	Exon 15	g.93597 C>T	cac**c**cag	p. T807T	ECL2	Silent variant; possible effect on exonic splice enhancers predicted
57	Exon 5	g.78881 C>T	cgaaatta**c**agtctggga	p. Q242X	ECL1	Nonsense mutation
	Exon 13	g.90996 C>T	tat**c**gac	p. R602X	ICL3	Nonsense mutation
29^g^	IVS20+1	g.107375 G>A	cag**g**taa	—	—	Splicing
	Exon 6	g.80326_80335 del	agaa**aataaacta**tca	p. K270Ifsx10	ECL1	Stop codon at position 279
45^g^	IVS21+5	g.111016 G>A	tca**g**tag	—	—	See below^d^
47^g^	IVS7+1	g.80884 G>A	cag**g**taa	—	—	Splicing

del=deletion; fs=frameshift; ins=insertion; IVS=intron; PTCH domains: ECL1=extracellular loop 1; ECL2=extracellular loop 2; ICL3=intracellular loop 3; TM2=transmembrane 2; TM11=transmembrane 11.

a Numbering based on GenBank genomic DNA sequence AL161729 available at http://www.ncbi.nlm.nih.gov/entrez/viewer.fcgi? val=AL161729.27. Mutations are corrected for SNPs using SNPs from blood samples (BCCs 22–39, 48, 49 and 57) and from SNP databases (BCCs 21, 40–47, 50–56, 58–60).

b Applicable to exonic variants only. Protein numbering based on GenBank accession number NP_000255.

c Effect of intronic mutations on splicing was predicted using software at http://www.fruitfly.org/seq_tools/splice.html; effect of exonic mutations on exonic splicing enhancers was predicted using software at http://rulai.cshl.edu/tools/ESE/.

d In sample 39, the genomic variation C113540T in exon 23 (c. 3944 c>T) was detected in tumour tissue but not in blood. As this is a common SNP, it was not included here, although may possibly represent a somatic mutation.

e Possible AG to CT tandem change of unknown cause. Splice-site prediction software failed to detect the wild-type splice acceptor site in intron 7 and, therefore, could not predict the effect of this variation on splicing. However, as both of these variations are in the intron 7 splice acceptor site, they are likely to have an effect on splicing.

f Splice-site prediction software failed to detect the wild-type splice acceptor site in intron 21 and, therefore, could not predict the effect of any of these variations on splicing.

g Patient nos. 29, 45 and 47 had previously been exposed to azathioprine, but not for 2 years before the removal of BCC (see table 2 for details).

**Table 4:** Comparison of the overall *PTCH* mutation frequency, exon/intron ratio and the distribution throughout the PTCH transmembrane protein.

	**Azathioprine exposed *n* (%)**		**Non-azathioprine exposed *n* (%)**	
Total BCC	39		21	
BCC with PTCH mutation	21		11	
Total PTCH mutations	27		17	
Intronic mutations	9 (33%)		6 (35%)	
Exonic mutations	18 (67%)		11 (65%)	
Distribution in PTCH protein	ECL2	6	ECL2	4
	ECL1	7	ECL1	1
	Carboxy-terminal	2	ECL3	1
	ICL3	1	ICL3	2
	TM2	1	TM2	1
	TM11	1	TM5	1
			ICL2	1

ECL1=extracellular loop 1; ECL2=extracellular loop 2; ECL3=extracellular loop 3; ICL3=intracellular loop 3; ICL2=intracellular loop 2; TM2=transmembrane 2; TM5=transmembrane 5; TM 11=transmembrane 11.
