# Supplementary material for: PTCH mutations in basal cell carcinomas from azathioprine-treated organ transplant recipients
Source: Br J Cancer. 2008 Oct 14;99(8):1276–84. doi: 10.1038/sj.bjc.6604665 (PMC2570526; doi:10.1038/sj.bjc.6604665)
Supplement: Supplementary Table 1 [file 6604665x1.doc]

Supplementary Table 1: primer sequences and PCR conditions for amplification of exons 2 – 23 of PTCH.

| **Exon/**  **fragment** | **Primer sequence**  **(5’ → 3’)** | **Amplicon length (bp)** | **Annealing temperature for PCR (°C)** | **MgCl_2_ conc. (mM)** | **Primer concentration (nM)** |
| --- | --- | --- | --- | --- | --- |
| 2 fragment A fwd | actcctcccttctgcttcgt | 231 | 66 | 1.5 | 400 |
| 2 fragment A rev | gggcgctcttaccttccac |
|  |  |  |  |  |  |
| 2 fragment B fwd* | gcgcgctgggattaaaagcagcgaac | 123 | 58 | 2.0 | 200 |
| 2 fragment B rev* | gcgcgcatctctatcaaccgcgagga |
|  |  |  |  |  |  |
| 3 fwd* | gcgcgctgagtttgcagtgattttgc | 291 | 58 | 2.0 | 200 |
| 3 rev | gccttacctgctgctcatta |
|  |  |  |  |  |  |
| 4 fwd | tgttctatcatttcgaatttgcac | 226 | 58 | 2.5 | 200 |
| 4 rev | aaagaagaggccatgcgtta |
|  |  |  |  |  |  |
| 5 fwd | taacctaacgcatggcctct | 225 | 58 | 2.5 | 200 |
| 5 rev | tcaaaactgaaatggaacaaaca |
|  |  |  |  |  |  |
| 6 fwd | cgatgcgtttagaaggctct | 296 | 58 | 2.5 | 200 |
| 6 rev | ttttgctctccacccttctg |
|  |  |  |  |  |  |
| 7 fwd | acaagcccttaatgcactgg | 267 | 58 | 2.0 | 200 |
| 7 rev | tggcttttgaggaaaggaag |
|  |  |  |  |  |  |
| 8 fwd | tgcttcctgggaatactgatg | 242 | 58 | 2.5 | 400 |
| 8 rev | ttgcataaccagcgagtctg |
|  |  |  |  |  |  |
| 9 fwd | gtcgaggcttgtggaagtgt | 279 | 59 | 2.0 | 200 |
| 9 rev* | gcgcgcagcaggagcagtcatggaaa |
|  |  |  |  |  |  |
|  |  |  |  |  |  |
| 10 fwd | tgttcggcttttgttctgtg | 243 | 59 | 2.0 | 200 |
| 10 rev* | gcgcgccatttgtcaacggacagcag |
|  |  |  |  |  |  |
| 11 fwd | ctgaattgcatctcgcatgt | 189 | 58 | 2.0 | 200 |
| 11 rev | tgatgtccccaaagctctct |
|  |  |  |  |  |  |
| 12 fwd | tctgccacgtatctgctcac | 274 | 60 | 2.0 | 200 |
| 12 rev | atgggatgctggaagtcagt |
|  |  |  |  |  |  |
| 13 fwd | gaggcatgttggtgacctct | 202 | 58 | 2.0 | 200 |
| 13 rev | ttctgcacccaatcaaaagg |
|  |  |  |  |  |  |
| 14 fwd | ccatttccctgtttcagca | 478 | 58 | 2.0 | 200 |
| 14 rev | agagccttaagttgtggcaga |
|  |  |  |  |  |  |
| 15 fwd | tgggagaacaacccctacaa | 502 | 58 | 2.0 | 200 |
| 15 rev | gttgaagctgaacacgcaaa |
|  |  |  |  |  |  |
| 16 fwd* | gcgcgctccttctggctgcgagttat | 314 | 63 | 2.0 | 200 |
| 16 rev | ccagtgccttaggtctccag |
|  |  |  |  |  |  |
| 17 fwd | tgtaatgctgtgcgaagctc | 335 | 63 | 2.0 | 200 |
| 17 rev | cagggaaggcacctctgtaa |
|  |  |  |  |  |  |
| 18 fwd | tcctaacctgtgcccttctc | 404 | 63 | 2.0 | 200 |
| 18 rev | ctccagaggcccagacataa |
|  |  |  |  |  |  |
| 19 fwd | cgaggacaccttagccctct | 285 | 60 | 2.5 | 100 |
| 19 rev | gccagaggaaatgggttgt |
|  |  |  |  |  |  |
| 20 fwd | accaggtgaagtccagcaac | 244 | 58 | 2.0 | 200 |
| 20 rev | ggcccaatcacaatgatttc |
|  |  |  |  |  |  |
| 21 fwd | tttgttcatttctggcgttg | 260 | 58 | 2.0 | 200 |
| 21 rev | caggaaacacagcattcagc |
|  |  |  |  |  |  |
| 22 fwd | cgtgctttgagctttgagtg | 379 | 66 | 2.0 | 200 |
| 22 rev | cactaccacggtgggaagac |
|  |  |  |  |  |  |
| 23 fragment A fwd | aaacccaaggagggaagtgt | 418 | 70 | 2.0 | 200 |
| 23 fragment A rev | gacagtcacggaggcagaag |
|  |  |  |  |  |  |
| 23 fragment B fwd | gttctcacaaccctcggaac | 365 | 60 | 2.0 | 200 |
| 23 fragment B rev | ctcctctttgcctggctcta |

* these primers have a 6 nucleotide GC clamp added to the 5’end . Addition of this clamp resulted in the generation of less complex melting profiles, and therefore increased the sensitivity of MCA for these exons.
